# Heparin and Progesterone Exert Synergistic Effects to Improve the In-Vitro Fertilization Rate of Bovine Sperm Bound to Oviduct Cell Aggregates from the Isthmus

**DOI:** 10.3390/vetsci9070372

**Published:** 2022-07-20

**Authors:** Mohamed M. M. El-Sokary, Seham F. Shehata, Karima G. M. Mahmoud

**Affiliations:** 1 Department of Theriogenology, Faculty of Veterinary Medicine, Benha University, Benha 13511, Egypt; 2 Veterinary Medicine & Food Security Research Group, Faculty of Health Sciences, Higher Colleges of Technology, Abu Dhabi 17155, United Arab Emirates; 3 Department of Animal Wealth Development, Faculty of Veterinary Medicine, Benha University, Benha 13511, Egypt; seham.shehata@fvtm.bu.edu.eg; 4 Department of Animal Reproduction & AI, National Research Center, Dokki, Giza 12622, Egypt

**Keywords:** bovine, oviduct, cell aggregates, fertilization, heparin, progesterone

## Abstract

**Simple Summary:**

After mating, sperm starts its long journey with the ultimate goal of fertilizing the oocyte. Inside the oviduct, sperm is attached to the surface of epithelial cells. The intact and good-quality sperm are elected and stored. Many infertility-related problems are related to the short life span of the sperm and/or the delay of the capacitation process of sperm attached to the oviduct. Thus, the elongation of the life span of the sperm would be very helpful in overcoming such problems. We hereby aimed to evaluate the fertilization ability of sperm that bind to cell aggregates oviduct (infundibulum-ampulla-isthmus), and assess the effect of heparin and or progesterone on the in-vitro fertilization ability of sperm co-incubated with isthmus cells aggregates. The sperm bound to isthmus aggregates improved the rate of in-vitro fertilization compared to infundibulum and ampulla. Moreover, pre-treatment of mature oocytes with heparin and progesterone plays a coactive role that improves the in-vitro fertilization of sperm bound to cell aggregates from isthmus. In conclusion, binding to isthmus aggregates improves the in-vitro fertilization of bovine sperm. Additionally, heparin together with progesterone, exerts a synergistic action that improves the in-vitro fertilizing potential of sperm attached to isthmus aggregates.

**Abstract:**

After the copulation process, some sperm start the long journey with an ultimate goal of fertilizing the oocyte. Inside the oviduct, sperm are stored, waiting for the ovulated oocyte where they bind to the apical surface of the oviduct cells, which in turn hold sperm to form a sperm nest. The essential functions of the sperm reservoir include attaching spermatozoa to oviduct epithelial cells, selecting intact, good-quality sperm with an end result of extending sperm life expectancy. The current study aimed to evaluate the fertilization ability of sperm that bind to cell aggregates from different parts of the oviduct (infundibulum-ampulla-isthmus), and to assess the effect of heparin and or progesterone (P4) on the in-vitro fertilization ability of sperm co-incubated with cell aggregates from the isthmus. In-vitro fertilization was identified as a cleaved oocyte to two cells or more. The sperm bound to cell aggregates from the isthmus improved the rate of in-vitro fertilization (48.09%) compared to aggregates from the infundibulum (36.90%) and ampulla (37.61%). Moreover, pre-treatment of mature COCs with heparin (40 μg/mL) and P4 (80 nanomolar) play a coactive role that improves the in-vitro fertilization ability of sperm bound to cell aggregates from isthmus (63.33%), compared to 42.61% in the absence of cells aggregates. In conclusion, binding to cell aggregates from isthmus improves the in-vitro fertilization ability of Bovine sperm. Additionally, heparin, together with P4, exerts a synergistic action that improves the in-vitro fertilizing potential of sperm attached to cell aggregates from the isthmus of the bovine oviduct.

## 1. Introduction

In order for sperm to engage in fertilization, it must pass a number of physiological obstacles until it reaches the oocyte. Compared to sperm waves that fail to enter the oviduct, sperm that reach the isthmus are thought to have a more significant percentage of normal morphology and zona pellucida binding capacity [1]. In vitro sperm binding to oviduct cell explants has been used to explore the sperm-oviduct relationship in various species, including bovine [2], swine [3] and equine [4]. The oviduct is believed to harbor sperm after semen deposition and before fertilization by a sophisticated mechanism. There is evidence from in-vitro studies that both oviductal cells and fluid maintain sperm viability in cattle, most probably by providing glycolyzable substrates [5].

Several studies showed that the binding of spermatozoa to oviduct epithelial cells results in extended sperm lifespan [4], improved fertilizing capacity [6], increased zona binding potential and sustained viability of spermatozoa [7]. The oviductal sperm reservoir selectively retains uncapacitated, morphologically normal sperm and suppresses chromatin changes [8]. Binding to oviduct cells is not only important for sperm survival, but also selects spermatozoa with superior fertilization competence [9]. Moreover, a beneficial effect on sperm motility has been demonstrated using oviduct epithelial cell-conditioned medium [10] and co-cultures of spermatozoa with oviduct epithelial cells [11].

Different models have been used to study sperm-oviduct interaction, including the monolayers of bovine oviduct cells [12] and oviduct cell explant [13]. Wide varieties of additives have been incorporated into media where sperm were allowed to bind oviduct explant, which eventually affects the sperm lifespan and fertilizing ability [14]. Among the additives, progesterone and heparin were investigated for their effects on sperm bound to oviduct cells, and their role in enhancing fertilization outcomes. Previous evidence reported that in swine, using progesterone (P4) at nano-grams concentrations induced sperm release from oviduct monolayer cells, and consequently improved the in-vitro fertilization rate [14]. In a related context, it has been proven that heparin plays an influential role in triggering hyper-activated motility [15] and sperm capacitation in bovine [16] and horses [17], in addition to sperm release from oviductal epithelial cell monolayers [18]. In the current study, the oviduct cell explants (aggregates) were used, since they facilitated figuring out how cells from different segments interacted with bovine sperm. The study aims to: (1) assess the fertilizing capacity of sperm bound to cell aggregates from various oviduct sections (infundibulum-ampulla-isthmus); and (2) investigate the role of heparin and/or progesterone in improving the in-vitro fertilization rate of bovine sperm bound to oviduct cell aggregates from the isthmus.

## 2. Materials and Methods

All chemicals and reagents for oviductal explants were obtained from Sigma Aldrich (St. Louis, MO, USA), unless otherwise stated. 

### 2.1. Experiment 1: Effect of Pre-Incubation of Sperm with Cell Aggregates from Different Oviduct Segments on In-Vitro Fertilization Rate

**Figure 1:**
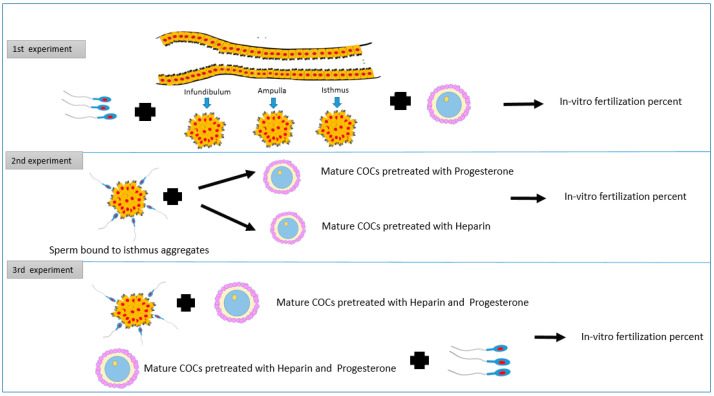
The study design shows three experiments. The first one evaluates the pre-incubation of sperm with cell aggregates from different oviduct segments on the in-vitro fertilization rate. The second experiment shows the impact of the addition of mature COCs treated with heparin or progesterone to sperm pre-incubated with isthmus cells aggregates on in-vitro fertilization outcomes. The third experiment aims to assess the influence of the addition of mature COCs treated with heparin and progesterone to sperm pre-incubated with isthmus cells aggregates on in-vitro fertilization outcomes.

This experiment aimed to investigate how pre-incubation of bovine sperm with cell aggregates from different oviduct segments affects the in-vitro fertilization rate (Figure 1).

**Figure 2:**
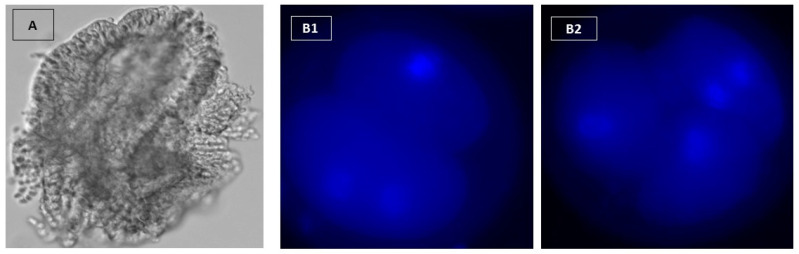
(**A**): Cell aggregates from oviduct with a circumference of 100–150 μm. Embryos were stained with Hoechst 33342. (**B1**,**B2**) show three cell-4 cell stages, respectively (X400).

The total number of oocytes used in this experiment was 663. The epithelium cell sheets from different parts of oviducts (infundibulum, ampulla and isthmus) were collected and processed further in dmTALP media to form aggregates. Frozen semen was allowed to bind the cell aggregates for 30 min at 39C. Afterward, mature COCs were introduced to the group of each segment, and free sperm as well (control). All groups were incubated for fertilization for 8–12 h under the same culture conditions (5% Bovine, 39 °C, 95% humidity). The rate of fertilization was then recorded for each group (Figure 2).

### 2.2. Experiment 2: Impact of the Addition of Mature COCs Treated with Heparin or Progesterone to Sperm Pre-Incubated with Isthmus Cells Aggregates on In-Vitro Fertilization Outcomes

**Figure 3:**
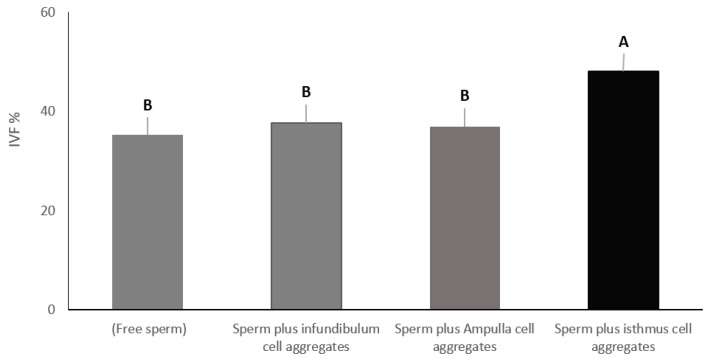
Cell aggregates from different oviduct segments (infundibulum, ampulla, and isthmus) were allowed to bind sperm. Mature COCs were added to all groups and co-cultured for in-vitro fertilization. Columns with different letters are significantly different (*p* < 0.01).

This experiment investigates the effect of heparin 40-μg/mL or progesterone at 80 nanomolar concentration on the in-vitro fertilization rate of sperm bound to cell aggregates from the isthmus. The total number of oocytes used in this experiment was 914. In group 1, mature COCs without any treatments were added to free sperm cells. In groups 2 and 3 we added mature COCs, pre-treated with progesterone at 80 nanomolar and heparin at 40-μg/mL, to free sperm cells, respectively. In groups 4 and 5, sperm cells were incubated with cell aggregates from the isthmus, then mature COCs, pre-treated with heparin at 40-μg/mL or progesterone at 80 nanomolar, were added to groups 4 and 5, respectively. Sperm cells were added to all groups at the same concentration, and all groups were incubated for fertilization 8–12 h under the same culture conditions (5% CO_2_, 39 °C, 95% humidity). The rate of fertilization was then recorded for each group. In this experiment, the oviduct epithelial cells aggregates were retrieved from the isthmus segment only, based on the findings obtained from the first experiment, and under the same circumstances (Figure 3).

### 2.3. Experiment 3: Influence of Addition of Mature COCs Treated with a Combination of Heparin and Progesterone to Sperm Pre-Incubated with Isthmus Cells Aggregates on In-Vitro Fertilization Rate

In this experiment, the effect of both heparin and P4 were investigated. The total number of oocytes used in this experiment was 589. In group one, mature COCs without any treatments were added to free sperm cells, while group two contained Mature COCs pre-treated with heparin 40 μg/mL plus progesterone at 80 nanomolar plus free sperm. Group three contained sperm cells incubated with cell aggregates from the isthmus plus mature COCs pre-treated with heparin 40 μg/mL plus progesterone at 80 nanomolar. Sperm cell concentration was the same for all groups. All groups were incubated for fertilization 8–12 h under the same culture conditions (5% CO_2_, 39 °C, 95% humidity). The rate of fertilization was then recorded for each group.

### 2.4. Collection of Oviduct Epithelial Cells

Oviducts were collected from entirely apparently normal pubertal females at a community abattoir, and transferred to the laboratory on ice. The oviduct’s infundibulum, ampulla and isthmus were trimmed rinsed in phosphate buffer saline (PBS), and the epithelial cell sheet was stripped in the lab. A glass microscope slide was used to apply pressure at a 45° angle. Epithelial sheets were collected in PBS and separated by centrifugation at 84× *g* for one minute in a 15-mL Falcon tube. After that, the supernatant solution was separated, and the cells were disaggregated 10 times with a 1000 μL pipette. PBS was added until the volume reached 15 mL, and the suspension was centrifuged for one minute at 84× *g*. The epithelial cells in the pellet were delicately passed 10 times via a syringe with a 22-gauge needle connected for accurate fragmentation. dmTALP medium was used to turn up the volume to 12 mL. In 100-mm cell culture dishes, the processed cells were split into three portions. Cells were incubated for 90 min at 39 degrees Celsius to allow them to re-aggregate. For sperm binding, spherical aggregates with a circumference of 100–150 μm were chosen [19].

### 2.5. Sperm Processing and Preparation

A pool of semen from three bovine bulls was used. Bovine sperm straws were thawed in a 39 °C water bath for one minute. We used a dm TALP medium at a pH 7.4 to assess sperm’s potential to bind cells from different sections of the oviduct (100 mM NaCl, 3.1 mM KCl, 1.5 mM MgCl_2_, 2.1 mM CaCl_2_, 0.29 mM potassium phosphate, 25 mM HEPES, 1 mM sodium pyruvate, 21.6 mM lactate, 6 mg/mL BSA, 100 U/mL penicillin and 100 mg/mL streptomycin). The sperm were separated by layering 50 µL of frozen-thawed semen under 2 mL medium, and allowing the spermatozoa to swim up during incubation for 1 h at 39 °C. After incubation, the top 1 mL from each tube was removed, pooled in a sterile 15 mL centrifuge tube, and centrifuged for 5 min at 600× *g* [20].

### 2.6. Assay of Sperm Binding to Oviduct Epithelial Cells

Spherical oviduct aggregates with a 100–150 micrometer circumference were washed two times in 100 µL drops of dmTALP media. Twenty oviduct cell aggregates were added to each sperm droplet. The final concentration of sperm in each droplet was 1.6 × 10^6^ cells/mL, and the assay was carried out in triplicates. Sperm and oviduct cell aggregates were incubated at 39 °C for 4 h to allow sperm binding. Gentle pipetting of sperm oviduct aggregates was applied to flush out and remove excess and loosely attached sperm. To confirm successful binding, images of sperm attached to oviduct cell aggregates were captured using a Zeiss Axioskop microscope [21]. For in-vitro fertilization experiments, the explants were gently washed to remove loosely attached sperm, and a new medium was introduced to flush out free sperm in the surrounding medium.

### 2.7. Oocyte Preparation

Briefly, the oocytes were placed in four well Petri-dishes containing 150 μL of TCM 199 medium (TCM-199 supplemented with 10% fetal calf serum, 50 µMcysteamine, and 50 µg/mL gentamycin sulfate). Each drop of media contained about 10–15 oocytes, covered with mineral oil, and incubated for 22 h at 5% CO_2_, 39 °C and 95% humidity [22].

### 2.8. In-Vitro Fertilization (IVF) and Culture (IVC)

Mature COCS were pre-treated, in dm TALP medium, with heparin and or progesterone at final concentrations of 40 μg/mL and 80 nanomolar, respectively. The mature COCs and sperm were then co-cultured for 8–12 h under the same culture conditions (5% CO_2_, 39 °C, 95% humidity). Then, the oocytes were washed in TCM-199 to remove the attached sperm, and vortexed for 2–4 min in a 1 mL centrifuge tube. Groups of 10–20 oocytes were placed in a pre-equilibrated 100 μL droplet consisting of TCM-199, 10% fetal calf serum and 50 μg/mL gentamycin. Pronuclei and cleavage rate were assessed after 48 h of culture, and the number of embryos was recorded using an inverted microscope [23,24].

### 2.9. Nuclear Staining of Embryos

The nuclear staining technique was carried out after Siqueira and Hansen [25]. By moving embryos from drop to drop, embryos were removed from the culture medium and washed twice in 100 μL droplets of PBS containing 1 mg/mL polyvinylpyrrolidone (PVP). Embryos were fixed in 100 μL droplets of paraformaldehyde [4% (w/v) in PBS, pH 7.4 for 1 h at 22 °C. Embryos were washed three times in 100 μL droplets of PBS-PVP and transferred to a 50 μL micro drop of 1 μg/mL Hoechst 33,342 for 10 min, and then washed two times. Embryos were placed in a small amount on a poly-l-lysine covered slide and left to dry at room temperature for 15 min. Circles were scribbled on the bottom of the slide with a diamond pen around the spot where the embryo was found. A minimal volume (2–16 μL) of antifade solution was applied to the area where the embryos were placed. After covering and drying for 2 h in a dark compartment, the cells were examined and counted by fluorescent microscopy (Figure 2B1,B2).

### 2.10. Statistical Analysis

Data were analyzed by ANOVA using Excel for Windows version 2019. Tukey’s multiple comparison tests were used to identify differences between means. Differences were considered significant at *p* < 0.05. The considered factors in the statistical analysis included: segments of the oviduct; sperm-explant co-incubation; and the effect of heparin and or progesterone.

## 3. Results

### 3.1. Experiment 1: Effect of Pre-Incubation of Sperm with Cell Aggregates from Different Oviduct Segments on In-Vitro Fertilization Rate

To evaluate the effect of pre-incubation of sperm with different sections of the oviduct on the outcomes of IVF, we designed four treatment groups. The first group included only free sperm (control). The second group included sperm plus cell aggregates from the infundibulum, the third group included sperm plus cell aggregates from the ampulla, and the fourth group included sperm plus cell aggregates from the isthmus. Sperm were added to all groups at the same concentration. Sperm and oviduct cell aggregates were incubated at 39 °C for 4 h to allow sperm binding. Then, mature COCs were added to all groups and co-cultured for 8–12 h under the same culture conditions (5% CO_2_, 39 °C, 95% humidity). The total number of oocytes that had cleaved to two cells or formed pronuclei were counted. In-vitro fertilization percentage in the group containing isthmus cell aggregates was significantly higher (48.09%), at *p* < 0.01, compared to the control group with free sperm (35.23%), the group containing ampulla cell aggregates (37.61%) and the group containing infundibulum cell aggregates (36.90%). On the other hand, there was no significant difference among the rest of the groups (control, infundibulum and ampulla) (Figure 3).

### 3.2. Experiment 2: Impact of the Addition of Mature COCs Treated with Heparin or Progesterone to Sperm Pre-Incubated with Isthmus Cells Aggregates on In-Vitro Fertilization Outcomes

**Figure 4:**
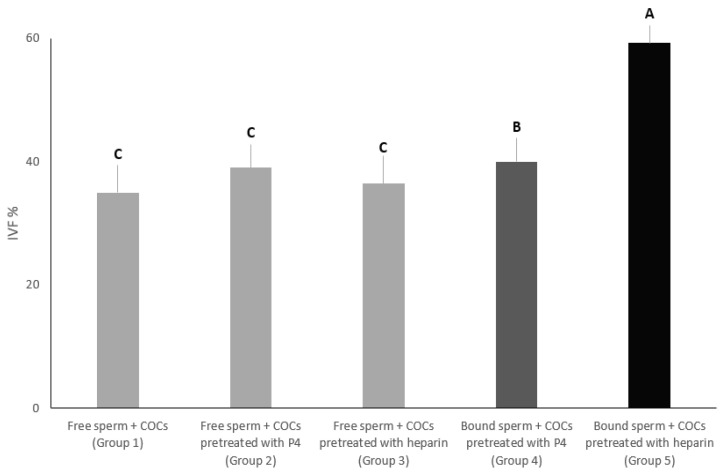
In group 1, mature COCs without any treatments were added to free sperm cells. In groups 2 and 3, mature COCs were added, pre-treated with progesterone at 80 nanomolar and heparin at 40-μg/mL, to free sperm cells, respectively. In groups 4 and 5, sperm cells were incubated with cell aggregates from the isthmus, and then mature COCs, pre-treated with heparin at 40-μg/mL or progesterone at 80 nanomolar, respectively. The in-vitro fertilization rate was recorded for all groups. Columns with different letters are significantly different (*p* < 0.01).

In this experiment, we aimed to evaluate the effect of progesterone at 80 nanomolar or heparin at 40-μg/mL on the in-vitro fertilization percentage of sperm co-incubated with cell aggregates from the isthmus. The percentage of oocytes that were fertilized in group 5 was significantly the highest (59.28%) compared to all groups (*p* < 0.01). On the other hand, the percentage of oocytes that were fertilized in group 4 was significantly higher (40%) than group 3 (36.42%), group 2 (39.04%) and group 1 (35%). Additionally, there was no significant difference among groups 3, 2 and 1 (Figure 4).

### 3.3. Experiment 3: Influence of Addition of Mature COCs Treated with a Combination of Heparin and Progesterone to Sperm Pre-Incubated with Isthmus Cells Aggregates on In-Vitro Fertilization Rate

**Figure 5:**
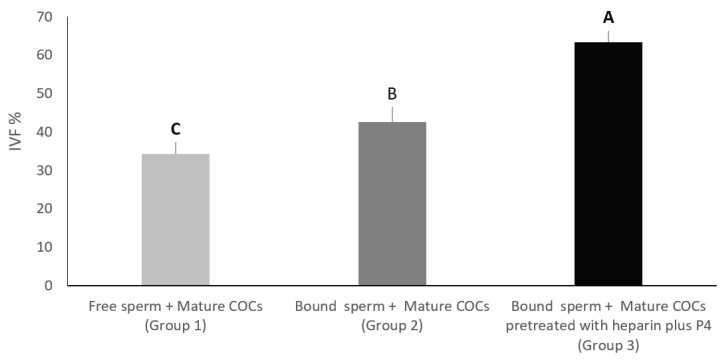
In group 1, mature COCs without any treatments were added to free sperm cells. In group 2, mature COCs pre-treated with heparin 40 μg/mL plus progesterone at 80 nanomolar, as well as free sperm. In group 3, sperm cells were incubated with cell aggregates from the isthmus plus mature COCs pre-treated with heparin 40 μg/mL plus progesterone at 80 nanomolar. All groups were incubated for fertilization. Columns with different letters are significantly different *p* < 0.05.

In this experiment, we wanted to evaluate the effect of the addition of mature COCs pre-treated with both heparin and progesterone on the in-vitro fertilization percentage of sperm pre-incubated with oviduct cell aggregates from the isthmus. In group one, mature COCs without any treatments were added to free sperm cells, while group two contained mature COCs pre-treated with heparin 40 μg/mL plus progesterone at 80 nanomolar plus free sperm. Group three contained sperm cells incubated with cell aggregates from the isthmus, plus mature COCs pre-treated with heparin 40 μg/mL and progesterone at 80 nanomolar. All groups were cultured for 8–12 h under the same culture conditions (5% CO_2_, 39 °C, 95% humidity). The percentage of oocytes that were fertilized in group three was significantly higher (63.33%) compared to group two (42.61%) and group one (34.28%), at *p* < 0.01. On the other hand, the percentage of oocytes that were fertilized in group two was significantly higher than that of group one (*p* < 0.05) (Figure 5).

## 4. Discussion

The goal of these experiments was to assess the fertilizing capacity of sperm that attach to cell aggregates from various sections of the oviduct (infundibulum-ampulla-isthmus) in the absence or presence of progesterone at 80 nanomolar and or heparin at 40-μg/mL. We found that sperm bound to the cell aggregates from the isthmus resulted in a higher IVF percentage compared to infundibulum or ampulla. The reason for the difference in fertilizing ability may be due to that bovine sperm has a high affinity toward the cell aggregates from the isthmus. This finding is in accordance with Elsokary [13], who reported that buffalo sperm have the highest affinity toward the cell aggregate retrieved from the isthmus.

Additionally, binding to cell aggregates from the isthmus not only may buffer the number of free sperm, but also can minimize polyspermy, which is consistent with Timothy [26]. In the same context, sperm attachment to isthmus cell aggregates may maintain low intracellular Ca^2+^, delay capacitation and prolong viability in sperm [27]. There is a scientific debate regarding which model to adopt for the effective study of sperm oviduct outcomes. Unlike using oviduct monolayer cell culture, our approach adopts cell aggregates as a model which simulates the real setting of oviduct epithelium, and are much easier to prepare and handle. Compared to the cell aggregates model, oviduct cells had been cultured for several days, and they appeared more flattened and fibroblast-like in their appearance [28]. In addition, their sperm-binding function changed during cultures lasting more than a few days [29]. Because the lifespan of capacitated sperm is short, regulation of the rate of capacitation of sperm stored in the isthmus could represent a crucial mechanism for ensuring the availability of viable sperm at the site of oocyte fertilization.

Our results show that the pre-treatment of mature COCs with progesterone at 80 nanomolar or heparin at 40-μg/mL improves the percentage of IVF significantly after allowing the sperm to bind cell aggregates specifically from the isthmus. On the other hand, the percentage of the IVF did not improve in the absence of cell aggregates from the isthmus, even if P4 or heparin were added individually. This means that binding to isthmus cell aggregates is necessary for enhancing the results of the IVF in bovine. The percentage of IVF increased significantly after mature COCs were pre-treated with progesterone at 80 nanomolar and heparin at 40-μg/mL, then added to sperm bound to cell aggregates from the isthmus. Moreover, when mature COCs were pre-treated with both progesterone and heparin, the IVF percentage increased compared to the control group. The reason for the improved IVF rate may be that heparin is crucial for capacitation and promotes sperm release from cell aggregates of the isthmus, which agrees with Ardon (2016) [30]. Additionally, using heparin at a concentration of 40 µg/mL resulted in an improved IVF rate in bovines [31]. It has been proposed that the glycans that bind to sperm are found on annexins [32]. Annexinsare also reported to contain heparin-binding sites. Thus, heparin may dislodge sperm from the oviduct by interacting with sperm receptors or oviduct cells [33,34].

Furthermore, the pre-treatment of mature COCs with P4 improves the IVF rate of bovine sperm bound to cell aggregates from the isthmus. The improvement of the IVF rate may be attributed to the effect of P4, which releases sperm from the aggregates. The sperm release is similar to the results obtained where the porcine sperm bound to cell aggregates from the isthmus [35], or SuleX-coated beads [36] were released after the addition of P4. Additionally, features were reported in swine, where P4 induced sperm release from oviduct monolayer cell culture and enhanced the in-vitro fertilization rate [12]. Saint-Dizier [37] suggested that the addition of P4 plays a crucial role in sperm release from the bovine oviduct epithelial cells (BOEC). At the physiological concentrations, P4 triggered in-vitro release of sperm cells, accompanied by a significant loss of surface protein (BSPs) and an escalation in membrane fluidity; both events possibly correlated to capacitation. Moreover, sperm cells bound to BOEC and then released by P4 presented significant phospholipid and protein structure changes.

## 5. Conclusions

In conclusion, binding to cell aggregates of the isthmus may improve the fertilizing capacity of bovine sperm in vitro. Moreover, at the time of fertilization, the addition of mature COCs, pre-treated with progesterone at 80 nanomolar and heparin at 40-μg/mL, to sperm bound to cell aggregates of the isthmus may exert a synergistic effect that improves the in-vitro fertilization rate of bovine sperm.

## Data Availability

The data analyzed for the study are available from the corresponding author upon reasonable request.
